# The effect of finerenone on arterial stiffness in patients with diabetic kidney disease: A pilot study 

**DOI:** 10.5414/CNP104S17

**Published:** 2025-11-28

**Authors:** Nejc Piko, Robert Ekart, Andrijana Koceva, Nika Alexandra Kravos Tramšek, Aleksandra Kukovič, Tadej Petreski, Luka Varda, Sebastjan Bevc

**Affiliations:** 1 Department of Dialysis, Clinic for Internal Medicine, University Medical Center Maribor,; 2 Faculty of Medicine, University of Maribor,; 3 Department of Endocrinology and Diabetes, Clinic for Internal Medicine, and; 4 Department of Nephrology, Clinic for Internal Medicine, University Medical Center Maribor, Maribor, Slovenia

**Keywords:** finerenone, chronic kidney disease, diabetes, arterial stiffness, atherosclerosis

## Abstract

Introduction: Patients with diabetic kidney disease (DKD) have an increased risk of not only renal, but also cardiovascular events. Finerenone is a novel, non-steroidal antagonist of the mineralocorticoid receptor that reduces albuminuria, protects kidney function, and improves cardiovascular outcomes. Materials and methods: Our aim was to evaluate the impact of finerenone on arterial stiffness parameters following 6 months of treatment. Additionally, we aimed to assess its effects on kidney function, potassium level, albuminuria (urinary albumin-to-creatinine ratio (UACR)), and hydration status, as measured by lung ultrasound (B-lines) and bioimpedance spectroscopy (BIS). Statistical analysis was conducted using SPSS. Results: We included 25 patients, with the average age 63.8 ± 8.4 years (range 43 – 73 years). Serum potassium increased after 1 month of treatment (4.5 ± 0.4 vs. 4.3 ± 0.4 mmol/L at baseline, p = 0.017) and remained stable afterwards. Kidney function remained stable, and UACR decreased slightly, with the lowest value at 3 months (67.0 ± 97.7 vs. 82.6 ± 116.6 g/mol at baseline, p = 0.054). Carotid-femoral pulse wave velocity decreased from 12.0 ± 3.1 m/s at baseline to 10.9 ± 3.1 m/s (p = 0.015). No effect on blood pressure or the degree of BIS overhydration was noted. However, we found a decrease in the number of B-lines after 6 months of treatment (4.9 ± 5.5 vs. 7.4 ± 8.4 at baseline, p = 0.021). Conclusion: Our findings indicate that after 6 months of treatment, finerenone led to a decrease in central arterial stiffness and interstitial lung water. Kidney function remained stable, no clinically significant hyperkalemia occurred.

## Introduction 

Diabetic kidney disease (DKD) is the primary cause of chronic kidney disease (CKD) and end-stage kidney disease (ESKD) globally. In 2022, 14% of adults aged 18 and older were diagnosed with diabetes, a significant rise from 7% in 1990. It is estimated that 30 – 40% of individuals with diabetes will develop DKD during their lifetime, underscoring the high prevalence of this condition [1]. 

Beyond the risk of ESKD, patients with DKD face significantly elevated rates of cardiovascular complications and mortality. The Action to Control Cardiovascular Risk in Diabetes (ACCORD) Trial found that DKD was associated with a 41% higher risk of cardiovascular events and a 39% increased risk of overall mortality compared to diabetes alone [2]. These findings emphasize the urgent need for new therapeutic approaches to mitigate cardiovascular and renal complications in this population [3]. 

Finerenone, the first novel non-steroidal mineralocorticoid receptor antagonist (MRA), has been developed with high potency and selectivity for the mineralocorticoid receptor (MR). Preclinical and clinical studies have demonstrated that finerenone reduces albuminuria, preserves kidney function, and improves cardiorenal outcomes. Notably, research in rats suggests that finerenone decreases central arterial stiffness, which may partially explain its protective effects on the kidneys and heart [4]. Given these findings, we have undertaken a prospective study to investigate the impact of finerenone on arterial stiffness in human subjects. 

## Materials and methods 

### Aim of the study 

The study’s primary objective was to evaluate the impact of finerenone on arterial stiffness parameters following 6 months of treatment. Additionally, we aimed to assess its effects on kidney function, potassium level, albuminuria, and hydration status, as measured by lung ultrasound and bioimpedance spectroscopy (BIS). 

### Inclusion and exclusion criteria 

Patients with albuminuric DKD who were prescribed finerenone, were included in the study. All patients agreed to participate in the study and signed an informed written consent prior to inclusion. Individuals aged less than 18, those with atrial fibrillation or aortic stenosis, as well as pregnant women and those with active malignancies at the time of the study were not included. Atrial fibrillation and aortic stenosis can affect peripheral pulse wave readings and arterial stiffness measurements, which is why patients with these conditions were excluded from the research [5, 6]. 

### Study design 

We conducted a prospective study involving 25 patients (64% male) with DKD who were prescribed finerenone (10 or 20 mg daily, based on prescription guidelines) at the outpatient Nephrology Clinic of the University Medical Center Maribor in 2024. The study protocol is outlined in Table 1. 

At the study’s initiation, we collected demographic data and anthropometric measurements. Throughout the follow-up period, patients underwent repeated blood and urine sample analyses. Kidney function was evaluated using the estimated glomerular filtration rate (eGFR), calculated with the Chronic Kidney Disease Epidemiology Collaboration 2009 creatinine equation (CKD-EPI 2009). Albuminuria was assessed through the urinary albumin-to-creatinine ratio (UACR) from a spot urine sample. 

Arterial stiffness was measured at baseline and after 6 months using noninvasive applanation tonometry (SphygmoCor, Atcor Medical, Sydney, Australia). Per prescription guidelines, finerenone dosing was adjusted based on eGFR and serum potassium levels, while all other prescribed medications remained unchanged during the follow-up period. 

Prior to inclusion, all patients provided written informed consent. The study received approval from the Institutional Ethics Committee (UKC-MB-KME/17-23) and was conducted in accordance with the Declaration of Helsinki and Good Clinical Practice guidelines. 

### Arterial stiffness measurements 

Two medical examiners conducted all the assessments. Each patient was instructed to rest in a supine position for 5 – 10 minutes prior to the measurements, and all electronic devices and mobile phones were turned off during the study to prevent interference with the data collected. Pulse wave analysis (PWA) was performed on the right radial artery. To be included, all measurements had to meet specific quality criteria: an operator index of ≥ 80%, average pulse height of ≥ 80%, pulse height variation of ≤ 5%, and diastolic variation of ≤ 5%. Pulse pressure (PP) was defined as the difference between systolic and diastolic blood pressure. The augmentation index (AIx) was calculated as the ratio of the augmentation pressure (AP) to PP, normalized to a heart rate of 75 beats per minute (AIx@75/minute) for uniformity across patients with different heart rates. Ejection duration (ED) was defined as the duration of left ventricular systolic ejection. The subendocardial viability ratio (SEVR) was calculated from the ratio of the diastolic to systolic time index. Carotid-femoral pulse wave velocity (cfPWV) was defined as the distance between the carotid and femoral arteries divided by pulse transit time (measured via electrocardiographic monitoring). Each patient underwent three optimal cfPWV measurements (with a standard deviation (SD) of less than 10%). The average cfPWV value was subsequently calculated and used as the study parameter. 

### Volume assessment 

To determine their volume status, lung ultrasound was performed using the LUST study protocol [5], and BIS (BCM, Fresenius, Bad Homburg, Germany) was performed at baseline and after 6 months. 

### Statistical analysis 

Statistical analysis was conducted using SPSS (Chicago, IL, USA). Basic descriptive statistics were applied to evaluate continuous variables (mean ± standard deviation), while categorical variables were expressed as frequencies and percentages. The Shapiro-Wilk test was utilized to assess the distribution of the variables. A paired samples t-test was employed to compare different normally distributed variables throughout the study. A p-value of less than 0.05 was considered statistically significant for all parameters. 

## Results 

The average age of the included patients was 63.8 ± 8.4 years, ranging from 43 to 73 years. The most prevalent comorbidities were arterial hypertension (n = 25, 100%), hyperlipidemia (n = 24, 96%), ischemic heart disease (n = 5, 20%), and heart failure (n = 4, 16%). A total of 11 patients (44%) were either current or former smokers. 

Finerenone was prescribed at a dose of 20 mg once daily for 14 patients (56%), while the remaining 11 patients (44%) received 10 mg once daily. Other commonly prescribed medications included renin-angiotensin-aldosterone system blockers (n = 23, 92%), statins (n = 23, 92%), sodium-glucose cotransporter-2 inhibitors (n = 19, 76%), diuretics (n = 18, 72%), acetylsalicylic acid (n = 15, 60%), and β-blockers (n = 13, 52%). Additionally, 20 patients (80%) were on metformin, 10 (40%) were receiving glucagon-like peptide-1 receptor agonists, and 9 (36%) were insulin-dependent. 

Table 2 presents anthropometric measurements and laboratory parameters, while Table 3 summarizes arterial stiffness measurements, lung ultrasound, and BIS parameters at baseline and after 6 months of treatment. 

## Discussion 

The findings of our study show that 6 months of treatment with finerenone did not lead to a reduction in blood pressure (measured both peripherally and with the help of applanation tonometry). The increase in serum potassium was observed, but was slight and clinically insignificant. Evidence suggests that a high plasma aldosterone concentration and MR overactivation play a pathophysiological role in the development of several unfavorable cardiometabolic and renal outcomes via the activation of pro-inflammatory and pro-fibrotic pathways [7]. Unlike steroidal MRAs such as spironolactone and eplerenone, finerenone’s strong selectivity and affinity for MR result in a significantly milder impact on serum potassium levels and blood pressure [8]. Finerenone showed no significant impact on various hydration status markers, except for a slight reduction in interstitial lung water after 6 months of treatment. This effect may be attributed to a mild diuretic action of the drug and/or its beneficial effects on the myocardium [9]. Since we did not conduct echocardiography or any other cardiac imaging, this finding remains hypothetical and requires further investigation in future prospective studies. 

The reduction in albuminuria was small, differing from previous studies that demonstrated finerenone’s effectiveness in lowering albuminuria by an average of 21 – 38% after 90 days of treatment [10]. Albuminuria is known to fluctuate significantly due to various factors such as blood pressure, dietary intake, timing of measurement, prior physical activity, and infections. This variability reduces its reliability as a marker for assessing treatment efficacy [11]. Moreover, the small number of patients included limits the interpretation of this finding and its clinical significance. 

Our study demonstrated a statistically significant reduction in cfPWV (a surrogate marker of cardiovascular risk) after 6 months of finerenone treatment. To our knowledge, this is the first clinical study to report this finding. Several landmark trials (Finerenone in Reducing Kidney Failure and Disease Progression in Diabetic Kidney Disease (FIDELIO-DKD), Finerenone in Reducing Cardiovascular Mortality and Morbidity in Diabetic Kidney Disease (FIGARO-DKD), and Finerenone in Heart Failure with Mildly Reduced or Preserved Ejection Fraction (FINEARTS-HF)) have confirmed the protective cardiorenal effects of finerenone, leading to approval of the drug for the treatment of albuminuric DKD and/or heart failure with preserved or mildly reduced ejection fraction [12, 13], but none of these trials studied arterial stiffness. Gil-Ortega et al. performed a preclinical study on Munich Wistar Frömter rats, a model of CKD. In their study, they compared 12-week-old rats with albuminuria treated with finerenone (10 mg once daily) to age-matched rats without albuminuria treated with a placebo. After 4 weeks, they noticed that mesenterial arterial stiffness was much higher in the control group, compared to the finerenone-treated group. Finerenone reduced the β-index, a measure of intrinsic vascular stiffness and this finding positively correlated with a reduction in albuminuria. Additionally, rats treated with finerenone had a higher elastin percentage in the vascular wall and also exhibited lower levels of oxidative stress markers [14]. Extrapolating preclinical models to real-life clinical data, we can conclude that finerenone shows promise in preventing and reversing functional and most likely structural vascular wall changes that are present in patients with diabetes and CKD [15]. Its utilization and diverse effects should be further investigated in broader patient groups, including those with non-diabetic CKD, to enhance both renal and cardiovascular outcomes. 

## Authors’ contributions 

N.P. conceived and designed the study. A.K., A.K., and N.A.K.T. helped with the inclusion of patients. N.P. and L.V. performed all the measurements in the study. N.P., L.V., and T.P. collected all the data and performed the statistical analysis. N.P. wrote the manuscript. N.P., R.E., A.K., A.K., N.A.K.T., T.P., L.V., and S.B. read and critically evaluated the manuscript and gave final approval for publication. 

## Funding 

There was no funding for this research. 

## Conflict of interest 

The authors declare no conflict of interest. 

**Table 1.** The design and timeline of the study.

Study parameter	Baseline	Month 1	Month 3	Month 6
Demographic data	x			x
Anthropometric measurements	x			x
Finerenone titration	x	x	x	x
Hemoglobin (g/L)	x	x	x	x
eGFR (mL/min/1.73m^2^)	x	x	x	x
Serum creatinine (µmol/L)	x	x	x	x
HbA1c (%)	x			x
Serum potassium (mmol/L)	x	x	x	x
UACR (g/mol)	x	x	x	x
Arterial stiffness measurements	x			x
Lung ultrasound	x			x
BIS	x			x

eGFR = estimated glomerular filtration rate; HbA1c = glycated hemoglobin; UACR = urinary albumin-to-creatinine ratio; BIS = bioimpedance spectroscopy.

**Table 2.** Anthropometric and laboratory data of included patients at different time intervals during the study (n = 25).

Parameter	Baseline (mean ± SD)	Month 1 (mean ± SD)	p	Month 3 (mean ± SD)	p	Month 6 (mean ± SD)	p
BMI (kg/m^2^)	31.0 ± 5.1	/	/	/	/	30.7 ± 5.0	0.227
Hemoglobin (g/L)	147.4 ± 22.4	149.9 ± 21.1	0.130	148.4 ± 23.7	0.541	147.9 ± 20.7	0.814
Serum creatinine (µmol/L)	103.0 ± 33.2	105.0 ± 35.7	0.555	100.7 ± 35.2	0.265	101.0 ± 36.3	0.407
eGFR (mL/min/1.73m^2^)	65.2 ± 20.7	63.9 ± 20.8	0.555	66.2 ± 20.5	0.396	65.8 ± 21.9	0.742
UACR (g/mol)	82.6 ± 116.6	76.8 ± 128.5	0.382	67.0 ± 97.7	0.054	70.9 ± 104.3	0.082
Serum potassium (mmol/L)	4.3 ± 0.4	4.5 ± 0.4	**0.017**	4.5 ± 0.4	**0.010**	4.6 ± 0.5	**0.004**
HbA1c (%)	7.2 ± 1.3	7.1 ± 1.3	0.565	6.8 ± 1.0	**0.037**	6.9 ± 0.9	0.250

BMI = Body Mass Index; eGFR = estimated glomerular filtration rate; UACR = urinary albumin to creatinine ratio; HbA1c = glycated hemoglobin.

**Table 3.** Arterial stiffness measurements, lung ultrasound, and bioimpedance spectroscopy parameters at baseline and after 6 months of treatment with finerenone (n = 25).

Parameter	Baseline (mean ± SD)	Month 6 (mean ± SD)	p
Central aortic systolic pressure (mmHg)	135.7 ± 19.5	134.7 ± 14.2	0.677
Central aortic diastolic pressure (mmHg)	88.1 ± 15.5	88.4 ± 11.3	0.856
SEVR (%)	148.2 ± 25.4	148.1 ± 26.8	0.984
AIx@75	28.4 ± 8.4	28.3 ± 7.1	0.926
AP (mmHg)	13.9 ± 7.6	13.3 ± 5.0	0.574
PP (mmHg)	47.2 ± 13.2	46.4 ± 9.0	0.690
ED (ms)	36.2 ± 3.7	36.2 ± 4.1	1.000
cfPWV (m/s)	12.0 ± 3.1	10.9 ± 3.1	**0.015**
B-lines	7.4 ± 8.4	4.9 ± 5.5	**0.021**
BIS - OH (L)	0.3 ± 1.5	0.0 ± 1.0	0.284

SEVR = subendocardial viability ratio; AIx@75 = augmentation index, normalized for heart rate 75/minute; AP = augmentation pressure; PP = pulse pressure; ED = ejection duration; cfPWV = carotid-femoral pulse wave velocity; BIS - OH = bioimpedance spectroscopy overhydration.
